# Preferences for accessing sexual and reproductive health information and services among adolescent girls and young women in higher learning institutions in Tanzania: A qualitative study

**DOI:** 10.1371/journal.pone.0352671

**Published:** 2026-07-10

**Authors:** Lusajo Jason Mwalukunga, Fabiola Vincent Moshi, Stephen Matthew Kibusi

**Affiliations:** 1 Department of Nursing Management and Education, University of Dodoma, Dodoma, Tanzania; 2 Department of Clinical Nursing, University of Dodoma, Dodoma, Tanzania; 3 Department of Public Health and Community Nursing, University of Dodoma, Dodoma, Tanzania; Jimma University, ETHIOPIA

## Abstract

**Introduction:**

Adolescence is a critical developmental stage characterized by significant physical, emotional, and cognitive changes, including sexual maturation. However, this population group faces challenges such as early sexual initiation, limited contraceptive access, high sexually transmitted infection risks, including HIV (with 43% of new HIV infections in Tanzania occurring among youth, with higher rates found among young women), and unintended pregnancies, negative outcomes of limited access to sexual and reproductive health (SRH) information and services. Social, cultural, and policy constraints restrict access to SRH services, highlighting the need to understand adolescents’ preferences to improve accessibility and health outcomes.

**Aim:**

The study aimed to explore the preferences for accessing sexual and reproductive health information and services among adolescent girls and young women (AGYW) in higher learning institutions in Tanzania.

**Methods:**

This qualitative descriptive study was conducted at two universities in Dodoma, Tanzania. Purposive sampling recruited 13 sexually active students aged 19–24. Data were collected through in-depth interviews conducted in Swahili, audio-recorded, and transcribed verbatim. Data saturation was reached at the 13th interview.

**Results:**

Young women reported different preferences for accessing sexual and reproductive health (SRH) information and services. They accessed SRH information through community, media, and healthcare-based sources. Family members,(particularly sisters)and friends, were key information sources, though parental reluctance limited access. Social media and search engines provided privacy but raised concerns about misinformation. Healthcare facilities were perceived as reliable yet unwelcoming, leading many participants to prefer over-the-counter services. Participants advocated for reliable online platforms to enhance the accessibility and accuracy of SRH information.

**Conclusion:**

This study highlights AGYW’s preferences for accessing SRH information, emphasizing digital platforms and peer education. Strengthening these approaches while improving the youth-friendliness of healthcare settings is essential for improving SRH outcomes among this population.

## Introduction

Adolescence stands out as a pivotal stage of development marked by intricate shifts in physical, emotional, and cognitive realms. Amidst this phase, sexual maturation takes center stage. Young individuals may encounter various hurdles such as premature initiation into sexual activity, insufficient availability of contraceptives, heightened vulnerability to sexually transmitted infections (STIs), including HIV, unintended pregnancies, and premature entry into parenthood [1]. Around 89% of the 1.8 billion individuals worldwide aged 10–24 live in developing countries [2,3]. According to the World Health Organization, roughly 21 million girls aged 15–19 in these regions become pregnant annually, with approximately 12 million giving birth [4]. These figures underscore the significant gaps in access to sexual and reproductive health services among young people.

The inadequate reproductive health outcomes observed among adolescents and young adults are linked to factors such as limited utilization of contraception and methods for preventing STI/HIV infection, physiological immaturity, insufficient access to reproductive healthcare services, and challenging socio-economic circumstances. Identifying the determinants that impact the utilization of sexual and reproductive health services among individuals aged 10–24 is crucial for devising successful programmes [5,6].

Research has consistently shown that young people have different needs and considerations across regions due to differences in demographics and social patterns, such as age, sex, urban or rural residence, marital status, education, employment, and sexual experience. These differences make AGYW more vulnerable than male youth and adult women [7,8].

AGYW encounter multiple barriers when seeking sexual and reproductive health services. Politically, laws and policies may discourage teenage sexual activity. Additionally, social, cultural, and religious influences have contributed to taboos surrounding discussions on teenage sexual activity, evident in the stigma associated with sexual health matters, particularly those concerning HIV/AIDS, family planning, maternal health, and sexually transmitted infections [9]. The negative consequences include 43% of new HIV infections in Tanzania occurring among youth below 24 years, with 70% of new adolescent infections occurring in girls, and 83% of the new infections in 2016 being in adolescent girls [10]. This indicates a need to understand the various preferences for SRH services that exist among AGYW.

### Theory of planned behavior

This study was guided by the Theory of Planned Behavior [11], which explains why people do or do not engage in certain behaviors. The theory postulates that the intention to perform a certain behavior can be predicted by three factors: attitudes toward the behavior, subjective norms, and perceived behavioral control.

Attitudes toward the behavior refer to the degree to which a person has a favorable or unfavorable evaluation or appraisal of the behavior in question. The second predictor is subjective norm, which refers to the perceived social pressure to perform or not to perform the behavior. The third antecedent of intention is perceived behavioral control, which refers to the perceived ease or difficulty of performing the behavior, reflecting past experience as well as anticipated impediments and obstacles.

As a general rule, the more favorable the attitude and subjective norm concerning a behavior, and the greater the perceived behavioral control, the stronger an individual’s intention to perform the behavior. In the context of this study, the Theory of Planned Behavior provides a framework for understanding how social norms, attitudes, and perceived control over health decisions shape AGYW’s preferences for accessing SRH information and services.

## Methods

### Study design and context

This study employed a descriptive qualitative case study design. The methodological orientation underpinning the study was thematic analysis, used to identify and interpret patterns within the data. According to Lambert, [13] a qualitative descriptive study draws from naturalistic inquiry, dedicated to examining phenomena as closely as feasible in their natural state within the confines of the research domain. The study aimed to explore the preferences for accessing sexual and reproductive health information and services.

The study was conducted in Dodoma, Tanzania’s capital city, at two university campuses: the University of Dodoma and St. John’s University of Tanzania, the only two universities in the Dodoma region as well as Tanzania’s central zone. The Dodoma region has a higher prevalence of risky sexual behavior, including multiple sexual partners (4.1%), intercourse with non-spousal partners (22.2%), and non-use of condoms (74.8%) [12].

### Sampling and participants

Purposive sampling was used to select both the universities and the study participants. A total of 13 participants were recruited; three additional eligible participants could not participate due to illness or emergency travel on the day of interview. Participant selection criteria included: being aged between 19 and 24 years, being enrolled in the second or third year of any degree programme, being sexually active, and having or having had multiple sexual partners.

### Data collection and analysis

Interviews were conducted by the principal investigator, a registered nurse with a Master’s degree in Nursing Education and a PhD candidate in Sexual and Reproductive Health. The researcher has prior experience in qualitative research. There was no prior relationship between the interviewer and participants. The researcher acknowledges potential bias due to prior assumptions about gaps in sexual health knowledge; however, efforts were made to minimize this through the use of a semi-structured interview guide and neutral probing techniques.

Topic guides were developed in English and then translated into Swahili to ensure consistency. The study was guided by the Theory of Planned Behavior [11] which informed the development of the interview guide and interpretation of participants’ attitudes and behaviors. Interviews explored societal stigma, cultural taboos, economic constraints, access to SRH information and services, and the scope of sexual and reproductive health education. All interviews were conducted privately within university premises, with audio recording and concurrent field note-taking. Interviews were conducted in Swahili and ranged from 25 to 80 minutes in duration.

Data saturation was determined by the research team, concluding at the 13th interview, with no interviews being repeated. The data collection process spanned one month from November to December 2023. Initially, screening questionnaires were distributed to all eligible students, requesting their mobile phone numbers for interview invitation purposes. Participants received financial compensation for their involvement; this information was withheld during recruitment to prevent bias and undue influence.

Data were analyzed using thematic analysis. Interviews were transcribed verbatim and read multiple times to ensure familiarity with the data. Coding was conducted manually without the use of qualitative data analysis software. Initial codes were generated inductively and grouped into categories, which were further organized into themes and subthemes. Themes were reviewed and refined to ensure consistency and accurate representation of participants’ perspectives.

### Trustworthiness

In qualitative research, “trustworthiness” denotes the extent to which data collected throughout the study are dependable, transferable, credible, and confirmable [14]. The trustworthiness of this study is described according to Korstjens and Moser [15].

Credibility refers to the assurance that research findings are accurate, derived from participants’ original data, and reflect their genuine opinions. Two strategies were employed: investigator triangulation and member checking. Investigator triangulation involved multiple researchers coding, analyzing, and interpreting the findings — two researchers collaborated, producing similar outputs. Member checking involved sharing results, interpretations, and conclusions with participants to enhance data robustness.

Transferability concerns the extent to which qualitative research findings can be applied to other contexts or settings. This study provides a comprehensive description of AGYW in higher learning institutions, including their behaviors, experiences, and contextual factors, thus enabling meaningful interpretation for individuals outside that context.

Dependability reflects the stability of findings over time, ensured through participant validation of findings, interpretations, and recommendations. Confirmability ensures that findings are not influenced by the researcher’s biases but are derived directly from the data. This study meticulously describes its steps up to the findings and adheres to established standards for qualitative descriptive study design during the analysis process.

### Ethical considerations

This study received ethical approval from the Institution Review Board of the University of Dodoma (Ref. MA.84/261/02/A/61/592). Permission to conduct the study was obtained from the Vice-Chancellor of the University of Dodoma and the Vice-Chancellor of St. John’s University of Tanzania.

Before each interview, participants received a briefing on the aim of the study, potential benefits and risks, the voluntary nature of participation, and conditions regarding anonymity and withdrawal. All participants provided written informed consent and verbal consent before recording. Confidentiality and privacy were maintained through the use of anonymized participant identifiers, masking of institutional data, and confidential identification numbers.

## Results

### Participant characteristics

A total of 13 unmarried adolescent girls and young women were interviewed: 6 were in the second year of study, and 7 were in the third year. The majority were in sexual relationships; their caregivers (head of family) had primary-level education, were self-employed, and had a monthly income below 350,000 Tanzanian Shillings. Most participants’ homes of origin were in rural areas; while at university, they resided on campus with education sponsored by the Higher Education Students’ Loans Board. Four themes were identified: community-based sources, media-based sources, healthcare-based sources, and diverse means for learning SRH information.

### Theme 1: Community-based sources

Sexual and reproductive health information was reported to be accessed through family members, especially sisters, and through friends, who were identified as major sources of information for AGYW.

#### Sub-theme I: Family members.

SRH information including information about family planning, menstruation, and sexual relationships was reported to be accessed through female family members, particularly sisters. Parents were described as a major obstacle to accessing this information. Fewer than a quarter of participants reported accessing information through family members; the majority found it difficult to approach family members.

*“About family planning, for example, I asked my sister, she is a nurse and was researching family planning among mothers in the community, so it was easy to ask her about those things.” (IDI #1, aged 23 years)*

*“Another way that we use is through my sisters who are experienced, so everyone will give and share what they know about something.” (IDI #7, aged 21 years)*

Parents were reluctant to share SRH information with their children, perceiving them as too young to know about these matters. The majority of respondents reported that parents became angry when asked about issues related to sex and SRH.

*“My parents, if you go and ask such things, they will be mad at you and think you have demons, that you have become a bad girl, and the world has changed you into a stupid girl.” (IDI #5, aged 22 years)*

#### Sub-theme II: Friends.

Over half of respondents identified friends as a major source of SRH information. Considering that parents were unwilling to share SRH information, participants found it more comfortable to ask their friends.

*“When I came here I met ladies who are experienced; they do sex for money, they go to night clubs, they go everywher, they are the ones who started to give me more information when I faced problems related to sexual relationships and everything.” (IDI #1, aged 23 years)*

*“Apart from social media, I ask close friends. I can read on social media and when I don’t understand I will ask my friend. But it is not easy for me to go to the hospital to ask the doctor about those issues. And I don’t think there is a large number of young women who do that.” (IDI #3, aged 23 years)*

Other studies also report that most young women prefer to access SRH information through their friends and some family members, feeling more comfortable with peers than with parents [16,17].

### Theme 2: Media-Based Sources

The majority of AGYW reported being more comfortable using media sources to access SRH information, as this guarantees their privacy. Media sources mentioned included social media platforms (Instagram and Facebook) and search engines (Google).

#### Sub-theme I: Sexual and reproductive health information accessed through social media.

Young women expressed that they find it more comfortable to access SRH information through social media. In-depth interviews revealed that AGYW spend considerable time on Instagram and Facebook to access SRH information.

*“My friends are experienced, and I’ve learned a lot from my friends, but also from social media or the internet. On the internet, I go just to confirm when my friend tells me I should use P2; I search on Google and I get information about P2, just like that. Although there are negative effects, it’s okay.” (IDI #1, aged 23 years)*

*“The internet and social media are my preferred ways of accessing information, but they have their challenges. You can go on social media and read that stuff, and mostly you end up reading things which are not important, and most of the issues there are not to be trusted one hundred percent.” (IDI #4, aged 21 years)*

Various studies report similar findings, with more than half of respondents citing the internet, especially social media, as a major means of accessing SRH information [16,17].

#### Sub-theme II: Sexual and reproductive health information accessed through search engines.

Search engines such as Google were reported to be one of the most important means of accessing SRH information. Participants expressed that they find it simple to get what they want by just googling.

*“I have a smartphone, therefore I use my phone to access information. If I decide to ask neighbors, you may find the information has spread all over the village. Therefore, most of the time I use my phone or even computer to access social media, I look, search, google for information that will help solve the problems I have.” (IDI #7, aged 21 years)*

*“I mostly use a smartphone; when I face challenges, I just google.” (IDI #4, aged 22 years)*

### Theme 3: Healthcare-Based Sources

#### Sub-theme I: Sexual and reproductive health services accessed through health facilities.

Participants reported accessing SRH services at health facilities, though some did so primarily because they had established relationships with specific doctors or nurses. While they recognized health facilities as providing reliable information, they reported that unfriendly healthcare providers and insufficient privacy created discomfort, leading them to seek services primarily when no alternative was available.

*“In the streets, there is no one who will go to the hospital. Or maybe if I can find my doctor, ask him to insert an implant, maybe that way.” (IDI #3, aged 22 years)*

*“Honestly, every one of us has a doctor. And we do all this because we are afraid of how people will judge us if they know that we have STIs or any other problems.”*

#### Sub-theme II: Sexual and reproductive health services obtained from over-the-counter shops.

Most young women reported preferring over-the-counter shops over formal medical institutions for obtaining SRH services, due to convenience, privacy, and the absence of judgmental attitudes. Without the need for appointments or communication with medical experts, over-the-counter shops provide a discreet and accessible way to obtain SRH products including pregnancy tests and contraception.

*“Speaking the truth I don’t get all the services at the hospital. For example, abortions are not conducted at the hospital; hospitals can’t help us to do abortions. Just like I said earlier, I can’t go to the hospital to ask for anything.” (IDI #1, aged 23 years)*

### Theme 4: Diverse Means for Learning Sexual Health Information

#### Sub-theme I: SRH education provided by peer educators.

Young women argued that SRH education should be provided in universities and led by peer educators. Peer-led initiatives create a safe and supportive environment where young women feel comfortable discussing sensitive topics related to their sexual and reproductive well-being. Peer educators, often close in age and sharing similar experiences, can offer relatable guidance and provide accurate SRH information.

*“There should be good counselors, girls who are our age, because girls in their first year come here at university and they don’t know anything about life at university. If they should create fliers, they should also prepare dialogues of discussion about these issues so that students can learn from one another.” (IDI #1, aged 23 years)*

*“For me, I think there should be a club, a health-related club here at university. We have an environment club, but I have never heard about a health club here. Having that club will be a good thing, and it will attract many students. So that club can organize seminars about these issues, I assure you more students will be ready to learn.” (IDI #8, aged 22 years)*

#### Sub-theme II: The need for a reliable online platform for SRH information.

The majority of AGYW recommended the development of a reliable online platform for SRH information, providing them with accessible and accurate information to support informed decision-making. Given that young people are the population group that utilizes the internet most, participants felt it would be helpful if SRH information on sensitive topics such as contraception, menstruation, STIs, and sexual relationships were available on platforms specifically designed for young women.

*“Also, there should be platforms that give this education to youths, for they need to know more issues like the consequences of abortion, unintended pregnancies, etc.” (IDI #5, aged 22 years)*

*“I can say technology can help, for example right now we can access issues like these through Google. We ask things there and we get answers, but it would be nice if we could have something more than just Google. Because through Google, you can search for information, but you can’t ask more questions or get in touch with the person who wrote that information. So, I think if there could be something that gives more options, you ask and you get answers instantly or even after a specified time.” (IDI #3, aged 22 years)*

## Discussion

This study aimed to explore AGYW’s preferences for sexual and reproductive health information and services. It builds on existing literature on the drivers of poor utilization of SRH services and provides insight for policymakers developing healthcare delivery systems that meet what AGYW need, both in higher learning institutions and in community-based settings.

### Community-based sources

The findings indicate that AGYW prefer to access SRH information through friends and certain family members. This preference arises in part due to significant barriers that limit open conversations within families, as the majority of parents and guardians are perceived as strict or unapproachable when it comes to discussing SRH. Despite being viewed as potentially reliable sources of information, many parents do not create an environment where such discussions feel safe. As a result, most young women turn to trusted friends, who they find more approachable and less judgmental. These results align with earlier studies: participants feel more comfortable asking experienced friends than approaching parents or healthcare specialists [16]. A study conducted in Kenya found that younger adolescents relied on their parents as a primary source of SRH information; however, some parents either avoided certain topics or provided insufficient detail [18]. Similarly, a study among young people in higher learning institutions in the Mbeya region, Tanzania, found that most participants reported difficulty discussing SRH matters with parents or guardians, preferring to talk to peers [19].

On the other hand, healthcare providers have influenced how AGYW prefer to access SRH services. Reports indicate that some providers use intimidating language, including remarks suggesting that infections are the consequence of frequent sexual activity [20]. According to the Theory of Planned Behavior, subjective norms — social pressures and influences — shape individual behavior [11]. The preference for family members and friends can be attributed to cultural and social norms in which young women feel more comfortable discussing sensitive topics with trusted individuals within their social circles. These findings imply that social influence from trusted sources should be integrated into SRH interventions.

### Media-based sources

AGYW indicated that the most common sources of SRH information were social media and search engines. These findings align with a study done in Kenya [18] which found that most participants relied on the internet for SRH information, most often to complement other sources. Participants stressed that technology, including internet search engines, social media, and mobile phones, has been a useful tool in meeting adolescents’ SRH information needs, as these options are convenient, available whenever needed, and enhance confidentiality. However, respondents noted uncertainty about the reliability of online information and expressed the desire for more credible platforms.

Findings align with a study by Patterson et al. [21] among adolescents aged 16–19 years, which found that participants preferred online platforms due to the challenges of accessing SRH information through cultural norms that perceive them as too young to discuss sexual health. These findings can be analyzed through the Theory of Planned Behavior: [11] the preference for media-based sources highlights how attitudes toward accessing SRH information are shaped by the perceived advantages of online platforms. Concerns about reliability, however, suggest that AGYW’s trust in these sources is not absolute. Perceived behavioral control influences their preference for online sources, as social media and search engines provide more autonomy; however, uncertainty regarding information credibility limits their sense of control over obtaining reliable information [22]. These findings emphasize the importance of developing reliable, evidence-based, and youth-friendly online SRH platforms.

### Healthcare-related sources

Most participants reported preferring over-the-counter medications over medical facilities when dealing with STIs or seeking contraception. The primary factor preventing this population group from favoring medical facilities was the lack of friendly healthcare providers. Participants also indicated that insufficient privacy created feelings of discomfort when discussing sensitive aspects of their sexual health, and they expressed concerns about being overheard. The Theory of Planned Behavior [11] provides a valuable framework for understanding these decisions. AGYW’s attitude toward over-the-counter medications is influenced by negative experiences and perceptions of formal healthcare settings, making over-the-counter options more appealing due to their perceived convenience and anonymity [23]. The findings align with a study done in Uganda, which showed that AGYW often lack information regarding available SRH services and face stigma and fear within healthcare settings [24]. The fear of judgment leads them to prefer over-the-counter options perceived as more accessible and less stigmatizing [25]. These findings underscore the urgent need to reform healthcare environments to be more youth-friendly, and to train healthcare providers to offer non-judgmental, confidential, and supportive care.

### Diverse means for learning sexual health information

Participants shared that peer education and reliable online platforms for SRH would help inform the majority of young women. Respondents also stated that university health clubs would be a preferred method for accessing SRH information. In line with other findings, university students reported learning more about SRH when health clubs were available on campus [17]. These findings can be explained by the Theory of Planned Behavior: AGYW’s attitudes toward peer education and online platforms are shaped by the perceived benefits of these sources as accessible, relatable, and capable of providing accurate information without the fear of judgment [26]. The perceived social pressures in many sub-Saharan African communities, where discussions about sexual health are often stigmatized, further lead AGYW to seek information from peers and online sources [27].

These findings imply a need to strengthen peer-led SRH education, expand digital health strategies, and institutionalize university health clubs as safe spaces for AGYW to access SRH information without fear of stigma. Universities and health organizations should prioritize training peer educators to enhance credibility and cultural acceptability. The reliance on smartphones and social media for SRH information suggests the necessity of developing more reliable online platforms and digital literacy programmes. Addressing social norms and stigma in SRH discussions is essential; community-wide interventions engaging parents, educators, and religious leaders can help normalize SRH discussions. Healthcare providers must ensure youth-friendly, confidential, and accessible SRH services by improving provider training, enhancing privacy measures, and integrating digital consultation options.

## Conclusion

This study offers valuable insights into AGYW’s preferences for accessing SRH information and services, emphasizing the influence of community-based sources, media platforms, healthcare-based sources, and various learning approaches. The findings reinforce existing literature on the barriers young women encounter in seeking SRH services, particularly highlighting the roles of social norms, provider attitudes, and privacy concerns in shaping health-seeking behaviors.

AGYW’s preference for discussing SRH matters with friends and select family members underscores the need to strengthen peer-led education programmes while fostering open conversations within families. The growing reliance on online platforms points to an urgent need for more credible, youth-friendly digital resources. Despite the benefits of digital platforms in ensuring confidentiality, concerns about misinformation highlight the necessity of regulating and improving the reliability of online health content.

Significant barriers within the healthcare system, including negative provider attitudes, lack of privacy, and fear of judgment, lead AGYW to opt for over-the-counter medications instead of formal medical services. Addressing these challenges requires targeted interventions, including training healthcare providers in youth-friendly service delivery, enhancing privacy measures, and integrating digital consultations. Institutionalizing university health clubs and structured peer-led programmes can create safe and supportive environments for young women to receive accurate SRH education. Policymakers should prioritize funding and policy reforms that strengthen peer education, expand digital health initiatives, and promote stigma-free healthcare environments.

## Supporting information

S1 Table Social demographic characteristics of participants (N = 13). (DOCX)

S2 Table Preferences of accessing sexual and reproductive health information and services among adolescent girls and young women in higher learning institutions. (DOCX)
