# Supplementary material for: Preferences for accessing sexual and reproductive health information and services among adolescent girls and young women in higher learning institutions in Tanzania: A qualitative study
Source: PLoS One. 2026 Jul 10;21(7):e0352671. doi: 10.1371/journal.pone.0352671 (PMC13354010; doi:10.1371/journal.pone.0352671)
Supplement: S1 Table — (DOCX) [file pone.0352671.s001.docx]

**S1 Table: Social demographic characteristics of participants (N=13)**

| **Variables** | **n (%)** |
| --- | --- |
| **Age** |  |
| - 19 to 21 | 2 (15.4) |
| - 22 to 24 | 11 (84.6) |
| **Institution** |  |
| - University of Dodoma | 8 (61.5) |
| - St. John’s University of Tanzania | 5 (38.5) |
| **Home residence** |  |
| - Rural | 5 (38.4) |
| - Small town | 4 (30.8) |
| - Big city | 4 (30.8) |
| **Caretakers** |  |
| - Biological parents | 9 (69.2) |
| - Guardians | 4 (30.8) |
| **Caretakers level of education** |  |
| - Primary | 2 (15.4) |
| - Secondary | 1 (7.7) |
| - College/University | 9 (69.2) |
| - No formal education | 1 (7.7) |
| **Caretakers’ occupation** |  |
| - Employed | 2 (15.4) |
| - Self employed | 11 (84.6) |
| **Family income level** |  |
| - Below 350,000 Tshs | 9 (69.2) |
| - Between 350 – 1,000,000 Tshs | 4 (30.8) |
| - Above 1,000,000 Tshs |  |
| **Birth order** |  |
| - First born | 7 (53.8) |
| - Last born | 3 (23.1) |
| - Others | 3 (23.1) |
| **Grew up with her siblings** |  |
| - Yes | 13 (100) |
| - No |  |
| **Belongs to a political party?** |  |
| - Yes | 3 (23.1) |
| - No | 10 (76.9) |
| **Do you consider yourself as religious person?** |  |
| - **Yes** | 13 (100) |
| - **No** |  |
| **Where do you stay?** |  |
| - In-campus | 11 (84.6) |
| - Off-campus | 2 (15.4) |
| **Education sponsorship** |  |
| - Higher Education Students loans board | 12 (92.3) |
| - Self-sponsored |  |
| - Others | 1 (7.7) |
| **Do you get financial support from your family?** |  |
| - Yes | 9 (69.2) |
| - No | 4 (30.8) |
| **Do you afford to possess valuable stuff like the latest version of mobile phones, laptops, etc?** |  |
| - Yes | 9 (69.2) |
| - No | 4 (30.8) |
