# Supplementary material for: Preferences for accessing sexual and reproductive health information and services among adolescent girls and young women in higher learning institutions in Tanzania: A qualitative study
Source: PLoS One. 2026 Jul 10;21(7):e0352671. doi: 10.1371/journal.pone.0352671 (PMC13354010; doi:10.1371/journal.pone.0352671)
Supplement: S2 Table — (DOCX) [file pone.0352671.s002.docx]

**S2 Table: Preferences of accessing sexual and reproductive health information and services among adolescent girls and young women in higher learning institutions.**

| **Theme Subtheme** | | |
| --- | --- | --- |
| Community based sources | | - Family members |
|  |  | - Friends |
| Media based sources | | - Social media |
|  |  | - Search engine |
| Healthcare Based Sources | | - Health facilities |
|  |  | - Over the counter shops |
| Diverse needs of services provision | | - Peer educators |
|  | | - Online platforms |
